# A Comparative Study of the Effect of Different Stabilizers on the Critical Quality Attributes of Self-Assembling Nano Co-Crystals

**DOI:** 10.3390/pharmaceutics12020182

**Published:** 2020-02-23

**Authors:** Bwalya A. Witika, Vincent J. Smith, Roderick B. Walker

**Affiliations:** 1 Division of Pharmaceutics, Faculty of Pharmacy, Rhodes University, Makhanda 6140, South Africa; bwawitss@gmail.com; 2 Department of Chemistry, Faculty of Science, Rhodes University, Makhanda, 6140 South Africa; v.smith@ru.ac.za

**Keywords:** nano co-crystals, crystal engineering, polydispersity index, zeta potential, particle size, zidovudine, lamivudine, HIV/AIDS, sonochemistry

## Abstract

Lamivudine (3TC) and zidovudine (AZT) are antiviral agents used orally to manage HIV/AIDS infection. A pseudo one-solvent bottom-up approach was used to develop and produce nano co-crystals of 3TC and AZT. Equimolar amounts of 3TC dissolved in de-ionized water and AZT in methanol were rapidly injected into a pre-cooled vessel and sonicated at 4 °C. The resultant suspensions were characterized using a Zetasizer. The particle size, polydispersity index and Zeta potential were elucidated. Further characterization was undertaken using powder X-ray diffraction, Raman spectroscopy, Fourier transform infrared spectroscopy, differential scanning calorimetry, and energy dispersive X-ray spectroscopy scanning electron microscopy. Different surfactants were assessed for their ability to stabilize the nano co-crystals and for their ability to produce nano co-crystals with specific and desirable critical quality attributes (CQA) including particle size (PS) < 1000 nm, polydispersity index (PDI) < 0.500 and Zeta potential (ZP) < −30 mV. All surfactants produced co-crystals in the nanometer range. The PDI and PS are concentration-dependent for all nano co-crystals manufactured while only ZP was within specification when sodium dodecyl sulfate was used in the process.

## 1. Introduction

More than 7000 people worldwide die of HIV-related causes daily. Many people are not benefiting fully from the use of orally administered antiretroviral (ARV) drugs, which provide the only effective means of halting the progression of HIV disease and AIDS [1]. Eight million of the estimated 37 million HIV-positive people should be treated, but only two million are currently receiving ARV therapy. This unmet need is expected to increase on an annual basis [1].

Crystal engineering is described as the exploitation of non-covalent interactions between molecular or ionic components for the rational design of solid-state materials [2,3]. The application of crystal engineering in pharmaceutics is usually related to understanding polymorphism and its associated properties.

Co-crystals are single-phase crystalline solids that are composed of two or more different molecules, which generally associate in a stoichiometric ratio [4]. Co-crystals can be constructed using several types of molecular interactions such as hydrogen bonds, halogen bonds, π–π stacking, van der Waal’s forces, amongst others [5,6,7,8]. They are thermodynamically more stable than crystals of the pristine compounds, while for pharmaceutical applications they are highly promising for tailoring the properties of the active pharmaceutical ingredient (API) [9]. Co-crystals are known to exhibit different properties from the parent compounds including enhanced solubility, improved dissolution kinetics, improved bioavailability as well as increased phase stability when compared to amorphous forms, which tend to spontaneously crystallize on standing. Co-crystal formation does not involve or require covalent bond formation or breaking and usually requires rather mild conditions during synthesis. Solid-state synthetic methods such as neat grinding, liquid-assisted grinding, and other mechanochemical methods have recently come into prominence as reliable methods for co-crystal synthesis and because they are inherently green methods capable of producing high yields without the need for large or excessive quantities of solvent [10].

Co-formers are molecules that are selected to co-crystallize with an API and are chosen from the, “generally regarded as safe” list (GRAS) or the, “everything added to food in the United States” list (EAFUS) [11]. They include but are not limited to food additives, preservatives, pharmaceutical excipients, and other API molecules [7,9].

Finally, co-crystallization of important API molecules may lead to patents or intellectual property protection emanating from their development [12].

Despite the advantages of co-crystallization, further benefit can be derived by combining different technologies to ensure targeted drug delivery, enhanced bioavailability, flexibility in respect of administration and stealth delivery. Combining co-crystallization with nano-sizing to yield nano co-crystals presents such an opportunity. Several techniques can be used to develop co-crystals with nano-scale dimensions, many of which are derived from techniques used in nanocrystal manufacture. Nanocrystals can be manufactured using two approaches, namely: a top-down technique that uses shear forces to reduce the particle size from micrometer to nanometer dimensions [13,14] and a bottom-up approach that involves nucleation and crystal growth. The growth of individual crystals can be arrested in the nanometer range by using a suitable stabilizer [15,16].

The use of surfactants as stabilizers has previously been explored in the synthesis of nanocrystals [17,18,19] and nano co-crystals [20,21,22]. Nano co-crystals are co-crystals of nano-scale dimensions which exhibit properties that are superior to those generally associated with co-crystals and nanocrystals [20,23,24]. Stabilizers are primarily used as growth prevention agents and function by the adsorption of surfactant/polymer molecules onto nucleated nanocrystals or co-crystals, lowering the surface free energy and consequently particle reactivity [25]. Known stabilizers include surfactants such as sodium dodecyl sulfate (SDS) [26,27,28], Tween^®^ [29,30,31], Span^®^ [20,32], α-tocopheryl polyethylene glycol succinate 1000 (TPGS 1000) [33,34], Pluronic^®^ [35,36] and polymers such as hydroxypropyl methylcellulose (HPMC) [19,37], pyrrolidone K30 [37] and polyvinyl pyrrolidone [19]. Different stabilizers impart different properties to the resultant nano co-crystals. For instance, TPGS 1000 is known to inhibit P-glycoprotein efflux and stealth properties to formulations in which it has been incorporated, while Tween^®^ 80 facilitates brain targeting [31,34,38,39] and Span^®^ is effective in reducing the size of nano co-crystals [20].

The use of a combination of techniques in the manufacture of nano co-crystals has been applied with success on a few occasions. For example, a top-down high-pressure homogenization technique (HPH) was used to produce nano co-crystals of the flavonoid, baicalein with nicotinamide. The resultant nano co-crystal exhibited a marked improvement in the rate and extent of dissolution [22]. Similarly, a bottom-up approach was used to develop myricetin-nicotinamide nano co-crystals. The nano co-crystal product also displayed an increased rate and extent for dissolution [40].

Sonochemical co-crystallization is a bottom-up process that has been successfully used for nano co-crystal synthesis [20,40,41,42]. One solvent systems involve dissolving all the co-crystal components in one solvent and injecting the solution into an anti-solvent while simultaneously sonicating the solution [41]. Two solvent systems involve dissolving the components of the co-crystal separately in different solvents followed by injecting each solution into the same anti-solvent at the same time. Top-down approaches such as wet media milling [40] and high-pressure homogenization [22], which are not covered in this work, have also been used with success.

Attempts have been made to produce co-crystals with nano-scale dimensions with varying success. The pharmaceutical compound caffeine and the co-former 2,4-dihydroxybenzoic acid were co-crystallized via sonochemical synthesis and stabilized with the surfactant Span^®^ 85. The resultant co-crystal dimensions for the smallest particles were 190 × 200 nm while the largest particles had dimensions of 200 × 800 nm [20]. The presence of surfactant was found to promote nucleation and moderate crystal growth. Myricetin-nicotinamide nano co-crystals were synthesised via both bottom-up and top-down approaches using Tween^®^ 80 as the stabiliser. The smallest particles had dimensions of 100 × 200 nm, whereas the largest particles were 200 × 800 nm [40].

Baicalein-nicotinamide (BE-NCT) nano co-crystals were successfully prepared via a top-down approach. The BE-NCT nano co-crystals were compared with BE coarse powder, BE-NCT co-crystals and BE nanocrystals, BE-NCT nano co-crystals exhibited a significantly enhanced performance both in in vitro and in vivo evaluations, suggesting that the nano co-crystals could be proposed as an advanced strategy for dissolution rate and bioavailability enhancement of poor soluble natural products such as BE [22].

Bhatt et al. synthesized a co-crystal of the ARV compounds 3TC and AZT using slow evaporation from a variety of solvents and several other methods including liquid assisted grinding [42]. The resultant co-crystal (3TC^.^AZT^.^H_2_O) contains a molecule of water, one molecule of 3TC and one molecule of AZT. Each AZT molecule hydrogen bonds via N–H^...^O and O–H^...^O interactions to three different 3TC molecules as well as to two different H_2_O molecules while each 3TC molecule hydrogen bonds to a single H_2_O molecule via an O–H^...^N interaction [43].

A preliminary investigation into the possibility of using a one or two solvent approach to producing nano co-crystals of 3TC and AZT proved unsuccessful. This was probably owing to the vastly different solubilities of the API molecules. This led to the development of a pseudo one solvent approach in which both components were dissolved separately in different solvents so that each solvent serves as an anti-solvent for the other, in situ.

Nano co-crystals are considerably easy to produce but stabilizing them against continual growth after forming and stabilizer(s) selection, are critical [44]. Herein, we report the use of surfactants in combination with sonochemical methods to synthesize nanometer sized co-crystals and to investigate the impact of different surfactants on the critical quality attributes (CQA) of the resultant nano co-crystal particles. To the best of our knowledge, this is the first comparative investigation of four stabilizers, viz., Tween^®^ 80, Span^®^ 80, SDS and TPGS 1000 and their effect on the three CQA parameters: particle size (PS), polydispersity index (PDI) and Zeta potential (ZP) for the reported co-crystals.

## 2. Materials and Methods

### 2.1. Materials

AZT and 3TC were purchased from China Skyrun Co. Ltd. (Taizhou, China). Tween^®^ 80, Span^®^ 80, SDS and TPGS 1000 were purchased from Merck (Johannesburg, South Africa). HPLC-grade water was prepared by reverse osmosis using a RephiLe^®^ Direct-Pure UP and RO water system Microsep^®^ (Johannesburg, South Africa) fitted with a RephiDuo^®^ H PAK de-ionization cartridge and a RephiDuo^®^ PAK polishing cartridge. The water was filtered through a 0.22 µm PES high flux capsule filter Microsep^®^ (Johannesburg, South Africa) prior to use. HPLC grade Honeywell Burdick and Jackson™ methanol (MeOH) was purchased from Anatech Instruments (Johannesburg, South Africa).

### 2.2. Methods

#### 2.2.1. Preparation of Micro and Nano Co-Crystals Using a Pseudo One Solvent Bottom-Up Method

Micro co-crystals of AZT and 3TC have been reported and were synthesized according to previously described methods [44]. The micro co-crystals produced were used as reference material in the characterization experiments in order to elucidate the characteristics of nano co-crystal formation in this investigation. A quantity of 3TC and AZT equivalent to 2 mmol of each ARV was accurately weighed using a model AG 135 Mettler Toledo (Greifensee, Switzerland) analytical balance and dissolved in 10 mL of water and 5 mL of ethanol (EtOH) respectively. The two solutions were mixed and gently stirred at 50 °C for an hour. The solution was allowed to cool to ambient temperature (22 °C) for 48 h to allow micro co-crystals to grow.

Supersaturation studies were conducted by adding 1 mL of MeOH to the 2 mmol of AZT and similarly, 1 mL of water was added to 2 mmol of 3TC. The individual solutions were then sonicated for 5 min using a Branson^®^ 8510E-MT ultrasonic bath (Danbury, CT, USA). Subsequently, 1 mL aliquots of solvent were added and further sonicated for 5 min until a clear solution resulted.

Nano co-crystals (NCC) of AZT and 3TC were prepared using a cold-sonochemical precipitation bottom-up technique [16,20]. The batch size was approximately 1 g, specifically 534 mg of AZT and 458 mg of 3TC amounting to the use of 2 mmol of each API. The 3TC was dissolved in 7 mL of water and AZT was dissolved in 6 mL of MeOH. The solutions were rapidly injected into a pre-cooled conical flask incubated at 4 °C ± 2 °C in an ice bath. A sonication output of 50 kHz ± 6 kHz was applied to the solution for 20 min using a Branson^®^ 8510E-MT ultrasonic bath.

#### 2.2.2. Particle Size Analysis

The mean PS and PDI of the NCC was determined using a Nano-ZS 90 Zetasizer (Malvern Instruments, Worcestershire, UK) with the instrument set to Photon Correlation Spectroscopy (PCS) mode. Approximately 30 μL of an aqueous dispersion of NCC was diluted with 10 mL HPLC-grade water prior to the analysis. The sample was placed into a 10 × 10 × 45 mm polystyrene cell and all measurements were performed in replicate (*n* = 6) at 25 °C at a scattering angle of 90°. Analysis of PCS data was undertaken using Mie theory with real and imaginary refractive indices set at 1.456 and 0.01.

#### 2.2.3. Zeta Potential

The ZP of the NCC was measured using a Nano-ZS 90 Zetasizer set in the Laser Doppler Anemometry (LDA) mode (replicates *n* = 6). The samples were prepared for analysis as described in Section 2.2.2 and placed into folded polystyrene capillary cells prior to measurement.

#### 2.2.4. FTIR Spectroscopy

The IR absorption spectrum of uncoated NCC was generated using a 100 Spectrum FTIR ATR spectrophotometer (PerkinElmer, Beaconsfield, UK) and analyzed using Peak^®^ version 4.00 spectroscopy software (Operant LLC, Burke, VA, USA). Approximately 5 mg of powder was placed onto a diamond crystal and analyzed over the wavenumber range 4000–650 cm^−1^ at a rate of 4 cm^−1^ (replicates *n* = 5) and the spectrum for the micro co-crystals was used for reference purposes.

#### 2.2.5. Raman Spectroscopy

The Raman spectra of uncoated NCC was collected using a Bruker Ram II spectrometer (Billerica, MA, USA) and analyzed using Peak^®^ version 4.00 spectroscopy software. Approximately 5 mg of material was placed into a stainless-steel cup and analyzed over the wavenumber range 4000–50 cm^−1^ (replicates *n* = 6) and the spectrum of the micro co-crystal was used for reference purposes.

#### 2.2.6. Differential Scanning Calorimetry

Approximately 4 mg of ultra-filtered and dried NCC was placed into aluminum pans and sealed. The pans were then placed directly into the furnace of a DSC 6000 PerkinElmer Differential Scanning Calorimeter (Waltham, MA, USA) and the data analyzed using version 11 Pyris^™^ Manager Software (PerkinElmer, Waltham, MA, USA). The temperature of the DSC was monitored with a computer and a controlled heating rate of 10 K/min was used for the analysis over the temperature range 30–150 °C. Thermograms were acquired at a rate of 10 scans/cm^−1^. All DSC analyses were conducted in triplicate (*n* = 3) under a nitrogen atmosphere purged at a flow rate of 20 mL/min and the thermogram for the micro co-crystal was used for reference purposes.

#### 2.2.7. Powder X-ray Diffraction (PXRD)

X-ray powder diffraction patterns were measured using a Bruker D8 Discover diffractometer (Billerica, MA, USA) equipped with a proportional counter, using Cu Kα radiation with a wavelength λ = 1.5405 Å and a nickel filter. All samples were placed onto a silicon wafer for the measurement of the diffraction pattern. The generator was set at 30 kV and the current to 40 mA. Replicate data (*n* = 3) was collected in the 2θ = 10 to 50° range at a scanning rate of 1.5 min^−1^ with a filter time-constant of 0.38 s per step and slit width of 6.0 mm. The X-ray diffraction data were treated using evaluation curve fitting (Eva) software. Baseline correction was performed on each diffraction pattern by subtracting a spline function fitted to the curved background. The diffractogram of the micro co-crystal was used for reference purposes.

#### 2.2.8. Energy Dispersive X-ray Spectroscopy Scanning Electron Microscopy

Energy-dispersive X-ray spectroscopy EDX, often also referred to as EDS, is based on the generation of X-rays following interaction of an electron beam with sample atoms. Apart from a continuous spectrum of X-rays generated by deceleration of beam electrons due to interaction with the atoms in a sample, sharp X-ray signals are produced at wavelengths that are specific for a given element. These signals form the basis for elemental mapping by SEM-EDX [45,46].

Elemental analysis was performed using a Vega^®^ Scanning Electron Microscope (Tuscan, Czechoslovakia Republic) fitted with an INCA PENTA FET. Approximately 1 mg of the NCC was dusted onto a graphite plate and the sample irradiated using SEM at an accelerated voltage of 20 kV (*n* = 3).

#### 2.2.9. Preparation of Surfactant-Coated Nano Co-Crystals via a Pseudo One-Solvent Bottom-Up Method

Surfactant-coated nano co-crystals were prepared as described in Section 2.2.1. Design Expert^®^ software version 8.0.71, Stat-Ease Inc. (Minneapolis, MN, USA) was used to generate experiments for a general factorial experimental design and the process factors investigated are listed in Table 1. The surfactants were added to the aqueous phase with the exception of Span^®^ 80, which was dissolved in methanol. The solutions were rapidly injected into a pre-cooled conical flask incubated at 4 °C ± 2 °C in an ice bath. A sonication output of 50 kHz ± 6 kHz was applied to the solution for 20 min using a Branson^®^ 8510E-MT ultrasonic bath (Danbury, CT, USA). The NCC suspension that was produced was characterized within 24 h of preparation. Characterization of the NCC included ZP, PDI, PS and FTIR, Raman spectroscopy, DSC, PXRD and EDX-SEM. The concentration of each of the surfactants was 0.5%, 1% and 2% *w/v* of the total volume used (Table 1).

## 3. Results

### 3.1. Co-Crystal Synthesis

The size of the co-crystals produced was smaller than that of the starting compounds when using the bottom up approach and the size reduction yielded sub-micron crystals. The uncoated micro cocrystals have a PS of 1593 ± 148 nm, PDI of 0.751 ± 0.063 and ZP of -6.86 ± 1.04 mV. The characterization of these crystals is reported herein.

### 3.2. Co-Crystal Characterization 

#### 3.2.1. FTIR Spectroscopy

**Figure 1:**
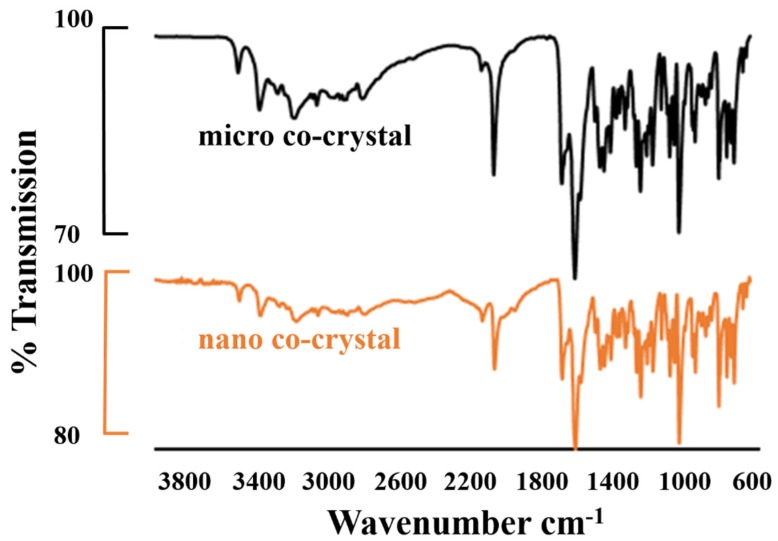
FTIR absorption spectra of the micro (black) and the nano co-crystal (orange).

The FTIR spectra depicted in Figure 1 have peaks at 3530 cm^−1^ for both nano and micro co-crystals, which is characteristic peak for water in the crystal structure [47,48]. The stretching band occurring at 1634 cm^−1^ is due to the carbonyl moiety (O=C–NR_2_) and is characteristic of 3TC. It partially overlaps with the band due to N–H bending at 1607 cm^−1^. The band at 1648 cm^−1^ is due to the stretching vibration of the imine group (R_2_-C=NR). Broad bands due to the stretching vibration of –NH_2_ and –OH functional groups are observed at 3300–3500 cm^−1^ and are indicative of 3TC in the co-crystal. Characteristic bands at 2170 cm^−1^ and 1652 cm^−1^ are due to –N_3_ and –N–H stretching vibrations and are indicative of AZT in the co-crystal.

#### 3.2.2. Raman Spectroscopy

**Figure 2:**
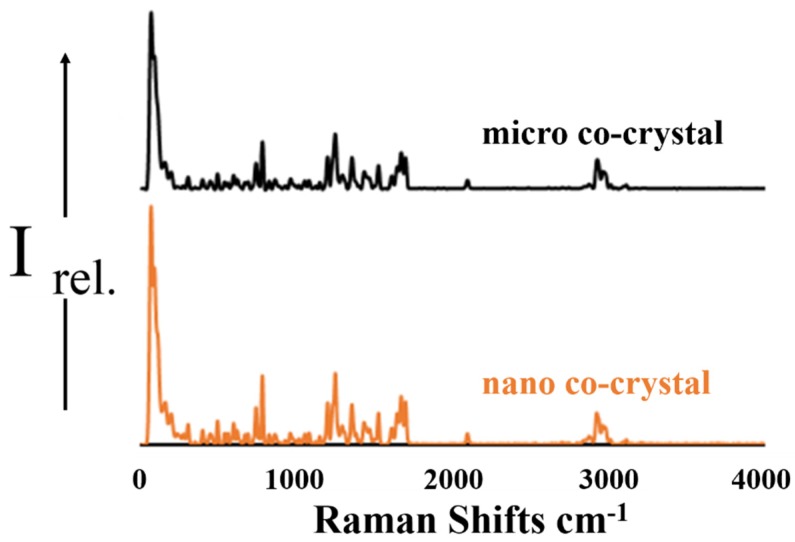
A stacked plot of the Raman spectra for the micro (black) and nano co-crystals (orange).

The Raman spectra illustrated in Figure 2 show characteristic peaks for both 3TC and AZT. In the region < 1500 cm^−1^ the Raman spectrum for 3TC exhibits several unique bands at approximately 1290 cm^−1^, 1250 cm^−1^ and 790 cm^−1^ [49]. A carbonyl stretching mode is observed at 1650 cm^−1^ in addition to C=N stretching at 1530 cm^−1^, both of which are confirmed in previous reports [49,50]. The most intense bands for AZT at 1650 cm^−1^ are due to the symmetric stretching vibration of the C=C bond of the pyrimidine ring. Another marker band for AZT is that corresponding to the breathing vibration of the pyrimidine ring located at 760 and at 790 cm^−1^. The peak at 1480 cm^−1^ is due to the carbonyl C=O stretching vibration of the pyrimidine ring. The band for the N≡N stretching vibration of the azide group present in the Raman spectrum at 2100 cm^−1^ is characteristic of AZT. The signals observed are in close agreement with previously reported data [51].

#### 3.2.3. Differential Scanning Calorimetry

**Figure 3:**
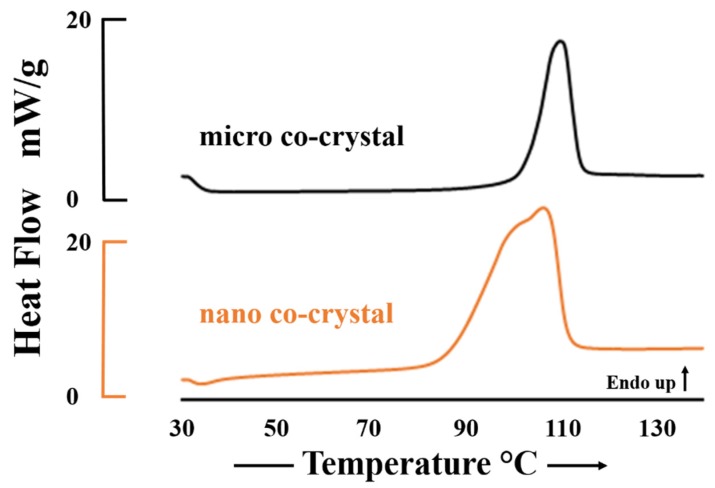
DSC thermograms depicting the melting endotherm for the micro (black) and nano co-crystal (orange).

The thermograms depicted in Figure 3 show the melting endotherms of the micro and nano co-crystalline material with T_peak_ = 106.7 °C and 103.1 °C respectively. The lower melting temperature and peak broadening for the nano co-crystal is likely due to particle size reduction and is in agreement with the principles of the Van’t Hoff equation [52,53,54]. In addition, slight peak broadening can be attributed to a loss crystallinity and partial formation of amorphous material during the formation of nanosuspensions [55,56].

#### 3.2.4. Powder X-ray Diffraction

**Figure 4:**
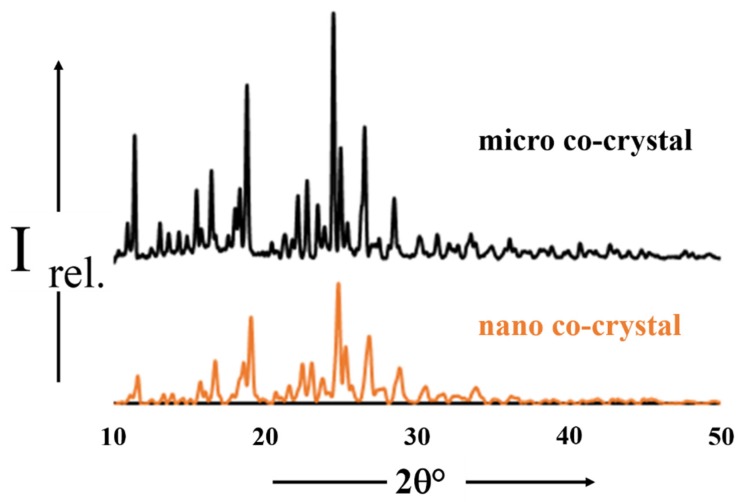
PXRD diffractograms for the micro (black) and nano co-crystals (orange).

It is apparent from Figure 4 that there is almost a one-to-one agreement between the diffractograms for the micro and nano co-crystal of 3TC^.^AZT^.^H_2_O. The only differences between the diffractograms relates to differences in the fine structure of the profiles. These differences may be attributed to particle size reduction of the NCC as the smaller the particle size results in broader peaks, which equates to less detail in the fine structure. Differences in the relative intensities of the peaks can be attributed to minor preferred orientation effects [57,58].

#### 3.2.5. Energy Dispersive X-ray Scanning Electron Microscopy (EDX-SEM)

The elemental composition of the nano and micro co-crystal indicates an identical percent composition of the elements present. The summary of elemental composition is summarized in Table 2. The data shows that the micro and nano co-crystal have the same elemental composition.

### 3.3. Surfactant-Coated Co-Crystals

Nano co-crystals were initially synthesized without the use of surfactants and then with surfactants to identify the effect of surfactant addition on crystal production. Generally, the use of surfactants yielded co-crystals in the nanometer size range. The trend observed shows optimal stabilization was achieved when electrostatic stabilization is prevalent. Non-ionic surfactants with high hydrophilic-lipophilic balance (HLB) values exhibited low steric stabilization concentrations due to the presence of relatively large hydrophilic heads in the surfactant. PS, PDI and ZP data for surfactant free NCC have been added to Table 3 for comparative purposes. The data reported for PS is intensity distribution and z-average.

#### 3.3.1. Particle Size (PS) and Polydispersity Index (PDI)

**Figure 5:**
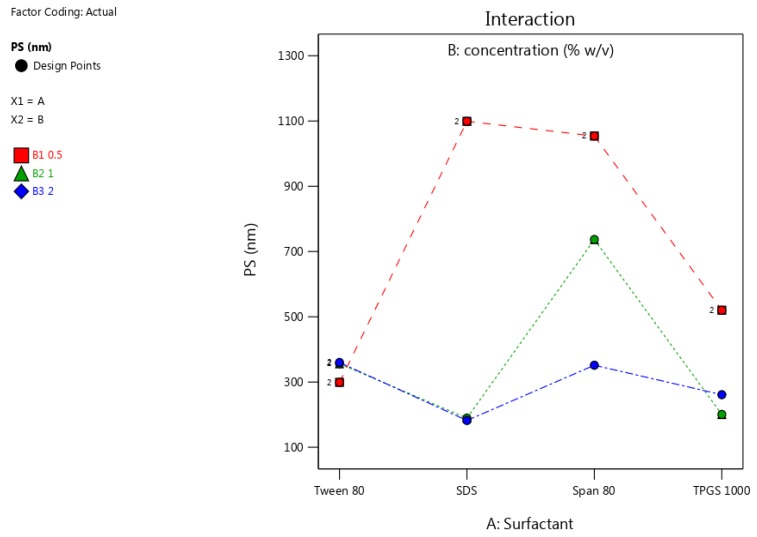
PS of nano co-crystals using different surfactants at concentrations of 0.5% *w/v* (red), 1% *w/v* (green) and 2% *w/v* (blue).

**Figure 6:**
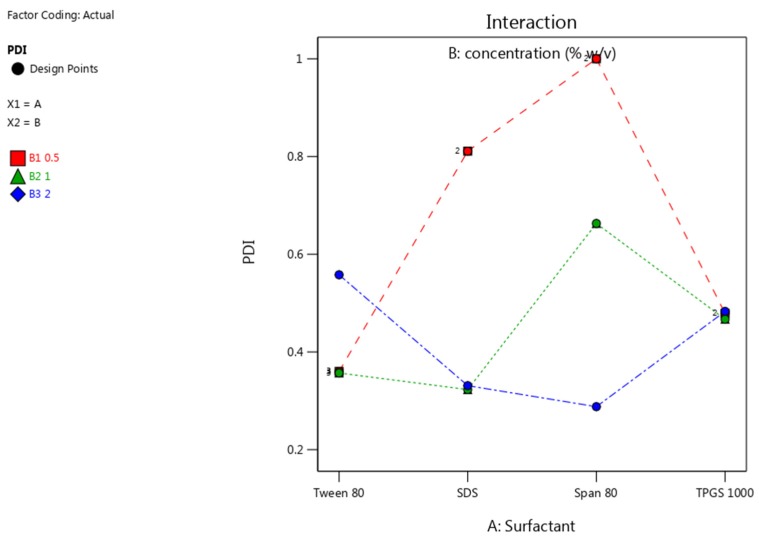
PDI of nano co-crystals using different surfactants at concentrations of 0.5% *w/v* (red), 1% *w/v* (green) and 2% *w/v* (blue).

The resultant particle size and PDI of the NCC was smaller when surfactant was used during the sonication phase of the production process. The interaction plots of PS and PDI using different surfactants and concentrations are depicted in Figure 5 and Figure 6.

The reduction in particle size observed was greatest when SDS was used, due the electrostatic stabilization achieved with this stabilizer. At lower concentrations, higher molecular weight surfactants exhibited sufficient steric stabilization. For instance, Tween^®^ 80 exhibited sufficient steric stabilization to produce crystals with a desirable PS and PDI, which can be attributed to the presence of a long hydrophobic chain and a considerably large hydrophilic head. It results in a reduction in PS and a low PDI indicating a narrow particle size distribution was achieved. The result is that fewer molecules of the surfactant are required to shield nucleating nano co-crystals from the balance of the materials in solution [25,59]. As the concentration of Tween^®^ 80 increased, the PDI and PS of the nano co-crystals increased, and this was attributed to agglomeration behavior of the crystals. Of the non-ionic surfactants used, TPGS 1000 produced crystals with the smallest size at intermediate concentrations with a PDI < 0.500 at all concentrations tested. The larger polyethylene glycol molecules have more polar heads and result in stabilization at lower concentrations due to a larger shielding effect while also forming a monolayer around crystals. The nano co-crystals stabilized using Span^®^ 80 exhibited a desirable PS and PDI as the concentration approached 2% *w/v*. Due to the presence of a smaller hydrophilic head, Span^®^ 80 requires a much larger concentration to achieve the same CQA when compared to TPGS 1000 and Tween^®^ 80. However, below the critical micelle concentration, ionic surfactants are better stabilizers than non-ionic compounds since ionic surfactants impart a surface charge to crystals, which results in electrical repulsion and better stabilization [60], as was evidenced for NCC stabilized using SDS.

The general trend depicted by the interaction plot for PDI in Figure 6 shows that an increase in concentration results in a lower PDI. As the concentration of each surfactant approached 2% *w/v* (blue line) the PDI values were lowest. This is attributable to the larg amount of available surfactant to prevent growth at the surface of several nuclei in solution resulting in a more uniform distribution of particle sizes. In addition to affecting the steric layer thickness, molecular weight and chain length; viscosity is also affected [60]. The higher the molecular weight, the higher the viscosity. In the final product, high viscosity can enhance the stability against aggregation [61]. This suggests that the molecular weight of the surfactants not only reduces particle or crystal size and PDI, but could potentially enhance stability.

#### 3.3.2. Zeta Potential

**Figure 7:**
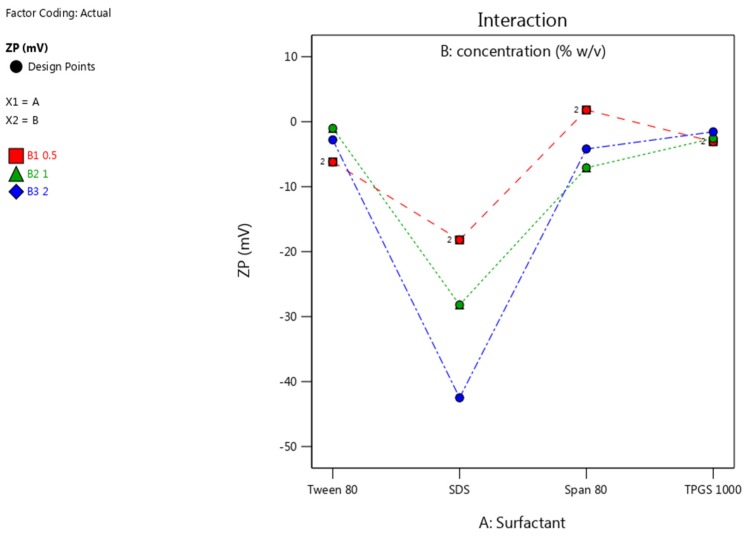
ZP of nano co-crystals using different surfactants at concentrations of 0.5% *w/v* (red), 1% *w/v* (green) and 2% *w/v* (blue).

The Zeta potential (ZP) was primarily affected by the presence of SDS, while the non-ionic surfactants resulted in a near neutral ZP. The rapid adsorption of negatively charged alkyl chains of SDS onto the surfaces of NCC, results in a negative charge at the surface [62]. The low ZP for the SDS stabilized nano-suspension follows the DLVO model [63]. The SO_4_^−2^ anionic head group of SDS is adsorbed onto the surface of the NCC, resulting in a negative charge at the inner Helmholtz plane (IHP). As adsorption continues, the Langmuir adsorption isotherm potential of IHP is increased and eventually leads to a lower ZP in the original dispersion medium, when compared to that in water [63]. The non-ionic surfactants offer no electrostatic stabilization and therefore the resultant ZP is close to neutral. It is therefore, expected that the nano co-crystals stabilized with SDS would exhibit better stability in solution when compared to NCC stabilized with non-ionic surfactants [64,65]. The impact of surfactant type and concentration used, on the ZP is shown in Figure 7.

## 4. Conclusions

3TC and AZT nano co-crystals were successfully prepared using a sonochemical synthesis approach. The use of different surfactants during the sonication phase of preparation resulted in the formation of crystals of reduced size, a reasonably narrow particle size distribution and in the case of the anionic surfactant, SDS, surface charge reduction. The use of surfactants to coat NCC may also achieve different purposes such as stealth and targeting capabilities, as these surfactant molecules are capable of interacting with specific substrates in biological systems [66,67,68].

All surfactants investigated exhibited concentration-dependent stabilization, resulted in the formation of nano co-crystals, and offered further stabilization characteristics. Stabilization was established to be concentration-dependent, based on the size of the hydrophilic head group of the surfactant used and surface charge induction. Surfactants with larger hydrophilic heads and thus larger HLB values, exhibited effective stabilization at lower concentration as observed by the ability of Tween^®^ 80 and TPGS 1000 to produce co-crystals in the nanometer size range when used at low and medium concentration.

The PDI of the NCC produced also exhibited a direct relationship to the concentration of surfactant used to achieve stabilization. It is clear that surfactants act as a steric barrier to crystal growth generated by precipitation and as a result, nucleation rate rather than growth rate increases with increasing surfactant concentration, resulting in smaller dimensions and narrow size distributions [69,70].

The use of the anionic surfactant SDS produced crystals with a low ZP. The contribution to ZP reduction appears to be an antagonistic concentration-dependent relationship with the anionic surfactant SDS. An increase in SDS concentration resulted in lower ZP which would, consequently, result in an expected increase in solution stability of the technology.

These results suggest that the use of any of these surfactants would result in the production of co-crystals in the nanometer size range, provided that the correct concentration of surfactant is used while only the surfactant SDS would produce nano co-crystals that meet all the CQA criteria set prior to commencing these experiments. The findings of this research could prove useful in overcoming the low bioavailability of AZT and potentially provide a dosage form capable of delivering AZT and 3TC to HIV reservoirs, thereby potentially reducing the side effect profile associated with ARV treatment. In addition, the potential to produce a long-acting and -circulating ARV regimen is a possibility.

Investigations into the use of combinations of surfactants for the production of nano co-crystals to evaluate the possibility of producing specific/targeted CQA in co-crystals is ongoing in our laboratory.

## Figures and Tables

**Table 1:** Summary of general factorial experiments.

Std. Run	Run No.	Surfactant	Concentration % *w/v*
8	1	TPGS 1000	1
2	2	SDS	0.5
11	3	Span 80	2
10	4	SDS	2
7	5	Span 80	1
5	6	Tween 80	1
9	7	Tween 80	2
4	8	TPGS 1000	0.5
12	9	TPGS 1000	2
3	10	Span 80	0.5
1	11	Tween 80	0.5
6	12	SDS	1

**Table 2:** Elemental composition of the micro and nano co-crystal.

Element	Micro Co-Crystal	Nano Co-Crystal
	Atomic %	Atomic %
C_K_	48.26 ± 0.52	49.67 ± 1.21
N_K_	20.75 ± 0.87	20.81 ± 0.94
O_K_	29.96 ± 0.73	28.53 ± 1.02
S_K_	1.03 ± 0.09	1.00 ± 0.03

**Table 3:** Summary of results from general factorial experiments.

Std. Run	Run No.	Surfactant	Concentration % *w/v*	PS nm	PDI	ZP mV
8	1	TPGS 1000	1	200.6 ± 28.91	0.467 ± 0.077	−2.57 ± 0.63
2	2	SDS	0.5	1099 ± 166.10	0.811 ± 0.051	−18.2 ± 2.35
11	3	Span 80	2	351.5 ± 21.19	0.288 ± 0.078	−4.2 ± 1.22
10	4	SDS	2	182.1 ± 11.60	0.331 ± 0.086	−42.5 ± 3.41
7	5	Span 80	1	736.7 ± 77.15	0.663 ± 0.022	−7.1 ± 1.13
5	6	Tween 80	1	356 ± 42.09	0.357 ± 0.008	−1.04 ± 0.35
9	7	Tween 80	2	360 ± 88.67	0.558 ± 0.093	−2.8 ± 0.12
4	8	TPGS 1000	0.5	520 ± 55.32	0.479 ± 0.072	−3.08 ± 0.95
12	9	TPGS 1000	2	261.2 ± 19.94	0.483 ± 0.043	−1.57 ± 0.22
3	10	Span 80	0.5	1054 ± 224.67	1.000 ± 0.000	1.8 ± 0. 84
1	11	Tween 80	0.5	299 ± 40.40	0.36 ± 0.089	−6.2 ± 1.98
6	12	SDS	1	189.3 ± 2.65	0.323 ± 0.094	−28.2 ± 4.61
N/A	N/A	N/A	N/A	1593 ± 148.32	0.751 ± 0.063	−6.86 ± 1.04
